# Evaluation of Masticatory Stimulation Effect on the Maxillary Transversal Growth in Ectodermal Dysplasia Children

**DOI:** 10.5005/jp-journals-10005-1408

**Published:** 2017-02-27

**Authors:** Elia Sfeir, Mona G Nahass, Ayman Mourad

**Affiliations:** 1 Professor, Department of Pediatric Dentistry, School of Dentistry, Lebanese University, Beirut, Lebanon; 2 Associate Professor, Department of Pediatric Dentistry, School of Dentistry, Lebanese University, Beirut, Lebanon; 3 Professor, Department of Mathematics, Faculty of Sciences, Lebanese University, Beirut, Lebanon

**Keywords:** Ectodermal dysplasia, Growth, Maxillary suture.

## Abstract

**Aims:**

Severe oligodontia is one of the most important symptoms in children with hypohidrotic ectodermal dysplasia (HED). The growth of the maxilla is a key consideration in restoring their mouth. The aim of this study was to evaluate the transversal maxillary sutural growth, after passive masticatory stimulation, in HED children. We also thought to assess the efficiency and functional outcome of the proposed propriocep-tive passive expansion (PPE) prosthetic device.

**Materials and methods:**

We studied 13 children (age 6-11 years) suffering from HED with severe oligodontia. Their maxilla was restored by a PPE device formed from two parts and joined by a passive slide system. Distance between the two parts was noted at the anterior and posterior regions at each control visit over an average of 23 months. We also conducted and filled a satisfaction questionnaire over the same period.

We tested the hypothesis that the posterior expansion is greater than the anterior expansion (one-tailed Student’s t-test with p-value <0.05). Best-fit linear and quadratic models were used to explore the relationship between age, duration of observation, and the rate of growth.

**Results:**

The average opening of the device was 2.27 mm in the anterior region and 2.96 mm in the posterior region. The questionnaire response was positive for all children. There are no significant linear or quadratic relationships between the data at the 5% significance level. The posterior expansion is greater than the anterior expansion at the 5% significance level (p-value 0.000394).

**Limitations:**

Further studies are mandatory to assess the reliability of our particular intervention and treatment modalities for these cases.

**Conclusion:**

The PPE device, we propose, assures function and esthetics in the long- term. It enhances stimulation by a passive way that leads to physiological growth of the palatal suture.

**Clinical significance:**

Using this PPE device to restore the maxilla in children with HED promotes physiological growth. The passive nature of this prosthesis helps by eliminating the need for any changes or replacement over time.

**How to cite this article:**

Sfeir E, Nahass MG, Mourad A. Evaluation of Masticatory Stimulation Effect on the Maxillary Transversal Growth in Ectodermal Dysplasia Children. Int J Clin Pediatr Dent 2017;10(1):55-61.

## INTRODUCTION

Ectodermal dysplasia (ED) is a hereditary genodermatosis characterized by a congenital defect of two ectodermal structures or more.^1^ Depending on the degree of the sweat gland dysfunction, ED is described as hidrotic or hypohidrotic ectodermal dysplasia (HED). The HED is the most prevalent form, with a frequency of 1 in 100,000 births, and its main characterized symptoms are hypohidrosis, hypotrichosis, and severe hypodontia.^2^ The literature describes a multitude of prosthetic treatments that enable functional, esthetic, and psychosocial rehabilitation of young patients with ED.^1,3,4^ Expert opinion concerning the traditional removable prosthesis in very young patients (3-5 years old) is unanimous.^3-6^ In fact, the oral rehabilitation of patients with partial or total prosthesis supported by the mucous membranes or the teeth (overdenture) is the most common and least expensive treatment modality.^5^ However, during this period, the maxilla is growing. Several theories have been proposed to explain this multifactorial orofacial growth phenomena.^7^ Several prosthetic options and follow-up have been proposed to support this growth.^8-10^

## AIMS

- To evaluate the transversal maxillary sutural growth, after passive masticatory stimulation, in children with HED.
- To assess the efficiency and functional outcome of the proposed prosthetic device.

## MATERIALS AND METHODS

A total of 21 children with clinical signs of ED were recruited from 13 families. Genetic examination (ORAgene DNA, DNA Genotek Inc., Ottawa, Canada) was conducted in collaboration with the Center for Dental Manifestations of Rare Diseases, Faculty of Dentistry, University Hospital, Strasbourg, France. It showed that 13 children were suffering from HED and 8 children from hidrotic ED with *WntlOA* gene mutation.^11^

We specifically studied 13 children suffering from HED between the age of 6 and 11 years (mean 8.6 years). All these children were boys with severe oligodontia. Only one of them has anodontia. Some of these children wore a removable prosthesis at a younger age.

**Figs 1A to D:**
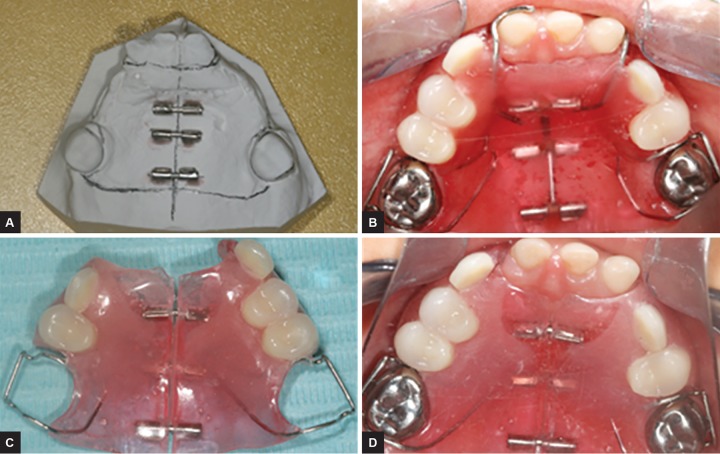
Steps of production of prosthesis with passive expansion: (A) Setting up the slides; (B) intraoral trying of the teeth assembly before separating the prosthesis into two parts; (C) prosthesis finished; and (D) prosthesis in the mouth

The study focuses on the possibility of growth of the maxilla after the age of 5 years, which is theoretically when the growth in this area ends.^7^ The mouth of these children has been restored with a “proprioceptive passive expansion” (PPE) removable prosthesis to (1) submit the maxilla to a passive stimulation during the mastication and (2) not interfere with the possibility of growth on the palatal suture. This appliance had an anteroposterior separation dividing it symmetrically into two parts (Figs 1A to D). These two parts are joined by a system of three passive slides. Each slide is formed by (1) two tubes cut from orthodontic bands (tube band HG 0.045-0.050 inch) and (2) an internal axis (wire 0.045 inch) (SR face bow ORMCO 1717, West Collins, Orange, CA, USA) (Figs 1A to D).

**Figs 2A to D:**
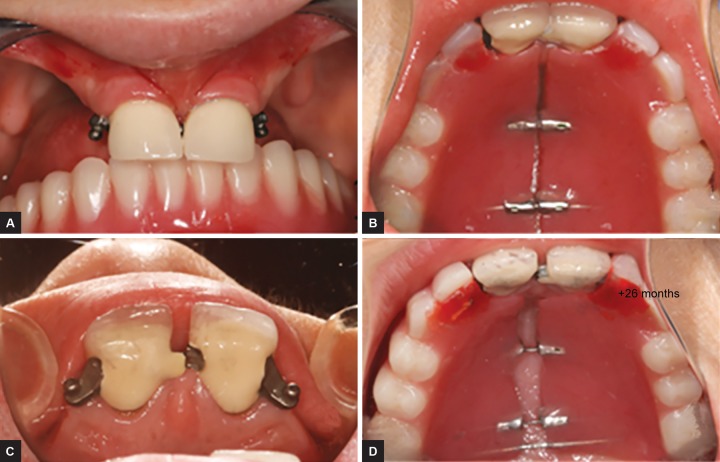
Result after 26 months, we can see the passive expansion of the denture and the opening of the slide between 11 and 21 allowing the accompaniment of sutural growth

**Figs 3A to D:**
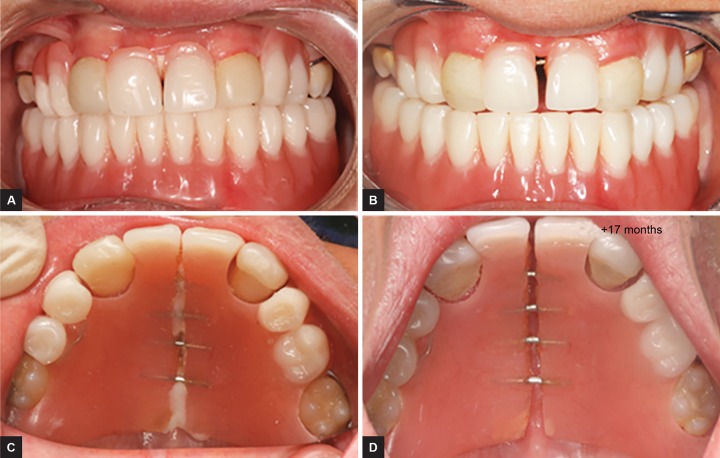
Evolution of growth after 17 months. This is one of the first case where four slides are used

Before setting up the final device, the first two models had some changes (Figs 2A to D and 3A to D), but they respond to the same principle of passive slide. At the mandible, a mini-implant retained over denture was performed.^4^

The width of the anteroposterior separation was measured using a digital caliper (Facom 1300PB, RCDE, Haute Garonne, France) on the day of placement of the device in the mouth at two levels: (1) The most anterior point, and (2) the most posterior point. The measures are rounded to the nearest half millimeter. Then, the same measures were repeated and recorded periodically during checkup visits. The observation period extended over an average period of 23 months.

In parallel, a satisfaction questionnaire was repeated at each control visit and included three questions: (1) “Does the prosthesis bother you?” (2) “Does it hold in your mouth?” and (3) “Do you get to eat with?”.

## STATISTICAL TEST

We tested the hypothesis that the posterior expansion is greater than the anterior expansion. To this end, we used the one-tailed Student’s t-test to compare the averages of the two measurements. A p-value <0.05 was considered statistically significant.

We used best-fit linear and quadratic models in order to explore the relationship between age, duration of observation, and the rate of growth.

## RESULTS

**Graph 1:**
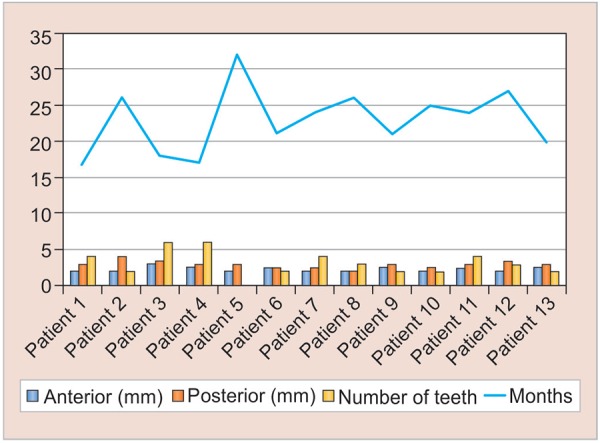
Number of teeth, duration of observation and expansion (anterior and posterior) for the 13 patients

Over an average period of 23 months, the average opening between the two parts of the prosthesis was of 2.27 mm (±0.33) in the anterior region and 2.96 mm (±0.52) in the posterior region (Graph 1).

The responses to questions 2 and 3 were positive for all children. For question 1, only three children complained from food impaction during meals.

**Graph 2:**
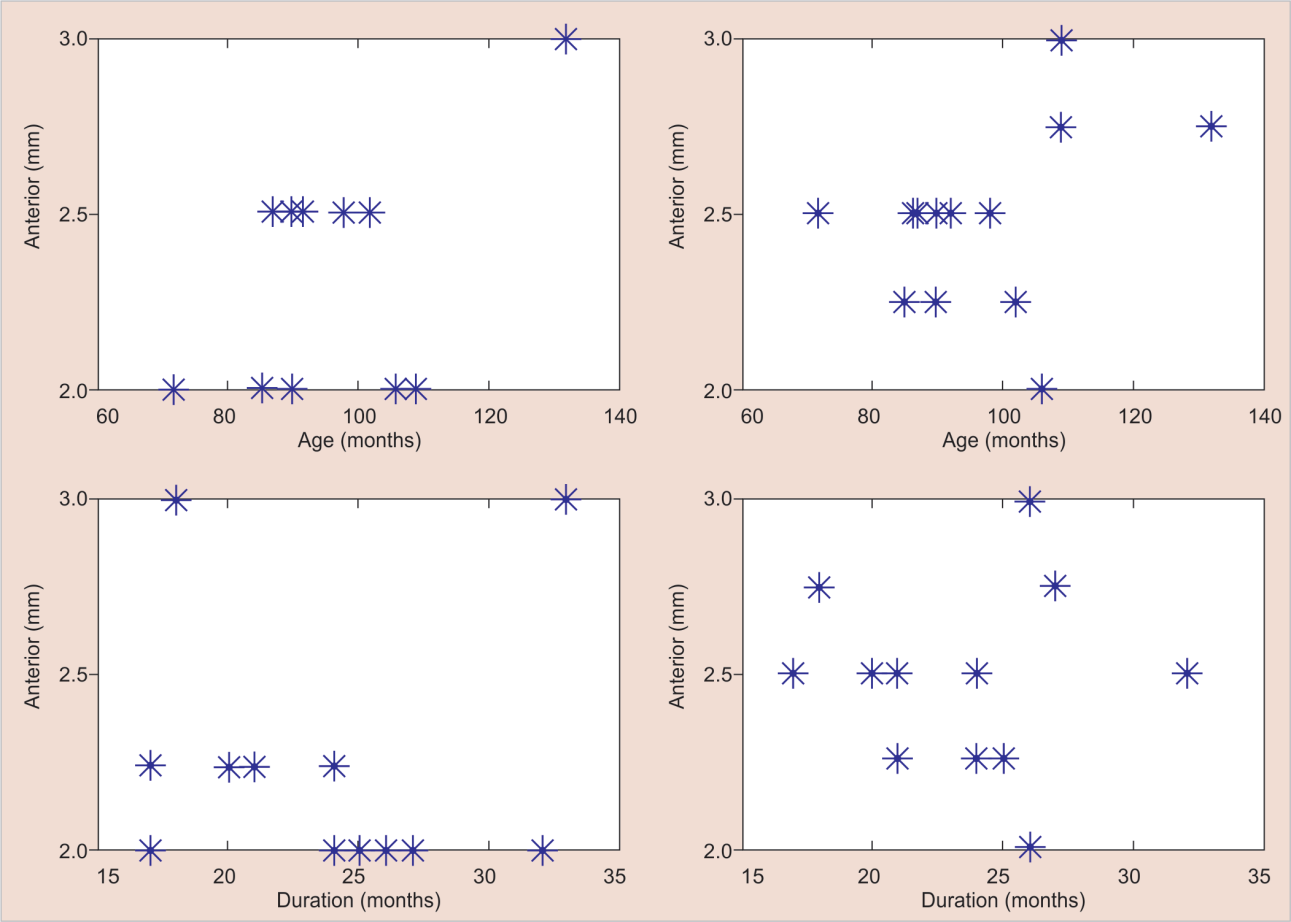
Illustration of the measurements

**Graph 3:**
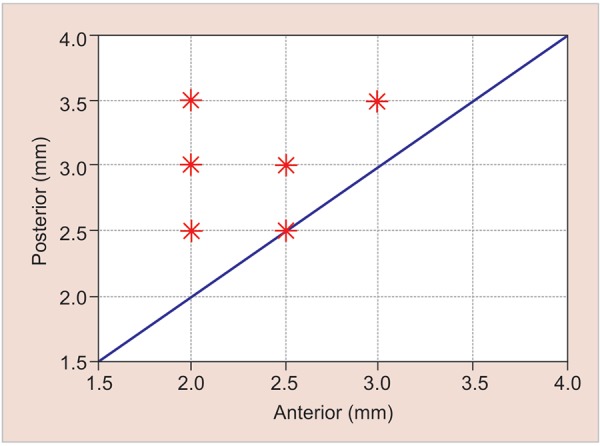
Comparison of the anterior and posterior expansions. The data of posterior expansion are all above the line, which means that they are greater than their corresponding anterior expansions

There is no significant linear or quadratic relationships between the data at the 5% significance level (Graph 2).The posterior expansion was greater than the anterior expansion at the 5% significance level. The p-value obtained from the one-tailed Student’s t-test was 0.000394 (Graph 3).

## DISCUSSION

To our knowledge, we report the first PPE device that permits the transversal growth of the maxilla with a device that accompanies passively the growth. The study focuses on children with HED and severe oligodontia (Graph 1). At the age studied, cases of ED children with *WNT10A* gene mutation possess almost all their primary teeth and, therefore, they do not require prosthetic restoration in the maxilla.^11,12^

It is generally accepted that in children with HED, prosthetic treatment must begin as soon as possible for reasons of self-esteem of the child and optimal psychological maturation. Authors agree that from the age of 3 to 5 years, the child begins to become sufficiently cooperating to accept such treatment. The total or partial resin denture is the most commonly used, and is satisfactory esthetically and functionally until a certain age.^8^ However, during this period, the maxilla is still growing. Several options have been proposed to adapt to this growth. Some authors recommend to change or modify the prosthesis every 2 or 3 months.^8,13,14^ For others, it is every 6 months.^15,16^ However, others propose observation on a year-to-year basis and recommend changing without much details.^10,17-20^ In 2012, Montanari et al^9^ provided a therapeutic approach by including a three-way screw in the prosthesis with an activation every 2 weeks. Unfortunately, regardless of the method used, there is always a risk of interference with the transversal growth of the maxilla given that the approach is empirical. Such an approach also remains dependent on the periodic visits of control by the patient.

In general, the maxillary growth is theoretically completed around the age of 5 years, but the palatal suture remains capable of opening up to the end of adolescence and catch up stunting.^21^ Several concepts are proposed to explain multifactorial orofacial growth phenomena.^7^ Although, for some authors, maxillary growth takes place even in the absence of teeth, it remains closely linked to the physiological stimulation of muscle function during chewing.^7,22,23^ We regularly observe, in cases of upper severe oligodontia, a transverse maxillary deficiency compared with the perimeter of the mandible. The methods proposed in the literature for mouth prosthetic rehabilitation in young children and the way of accompanying the growth may, in our opinion, lead to a reduction or exaggeration of the opening of the maxillary suture. As they are intermittent and do not respond to any biological criteria, they can expand the maxilla into an unstable (imbalanced) position.^7^ The device that we propose allows liberation of the palatal suture from any active prosthetic constraint. Thanks to the passive physiological stimulation of growth during mastication, the jaw can grow in the transversal direction without any prosthetic interference. It should be noted that the prosthesis we propose has been implemented in the majority of the cases after the age of 7 years, 2 years after the end of the maxillary growth. Without any changes to the device for an average of 23 months, it has, in some way, stimulated the growth and helped make up for lack of transversal growth of the maxilla.

**Figs 4A to D:**
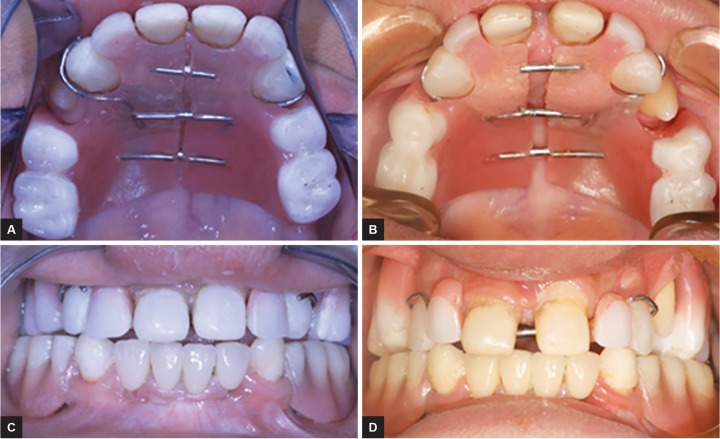
The 3 mm transversal growth (anterior) and 3.5 mm (posterior) after 18 months. The measures were performed before the repair of a fracture of the resin to the anterior region. Note the addition of composite on the occlusal surfaces of molars to make up for the vertical growth of the jaws

Our results show that the subsequent growth in the posterior region is slightly greater than that of the front region (Graph 1). Our results point in the same direction as those of Shirakawa et al^24^ or Tocchini et al,^25^ and are more consistent with that of Bhalla et al,^23^ if we consider the same age period studied. We advance that in these children, such a device can compensate for the lack of genetically programmed physiological growth even after the end of the maxillary sutural growth. The case in Figs 4A to D illustrates how the interrelationship between maxilla/mandible became normal after 18 months, thanks to the transversal growth of the maxilla. The lack of correlation between the age, duration, and the rate of growth can be clinically explained by the fact that this device, which stimulates the muscle function, allows the achievement of growth sutural, if this one is not completely finished.

However, the comparison between the posterior and the anterior measurements has shown that, in all of the cases, the posterior expansion is always greater than or equal to the anterior expansion. However, there is no relationship that allows predicting proportion of the anterior expansion from the posterior expansion and vice versa. For example, for the anterior expansion of 2 mm, the corresponding posterior expansions range from 2 to 3.5 mm (Graph 3).

To be noted that the questionnaire responses lead us to say that this type of device ensures the objectives sought by all prosthetic reconstructions, namely esthetic and functional, are met without having to periodically change the prosthesis. For the three cases where children have complained of food impaction, the solution was to reline, each time when it is necessary, the intraback of the prosthesis with a tissue conditioner (Coe comfort GC Corp, Bunkyo-ku, Tokyo, Japan). This relining does not interfere in any circumstances with the movement of the two parts of the prosthesis. After a period of 6 months, when we see that the slides do not open any more, intraback two parts of the prosthesis is sealed with a hard self-curable resin and transformed into a conventional prosthesis.

The HED is a very rare disease. In this article, the number of studied cases is limited. Therefore, future investigations with larger sample sizes are required. Further study designs are mandatory to assess the reliability and treatment modalities for these cases.

## CONCLUSION

In conclusion, we can say that masticatory stimulation is an important factor in the maxillary transversal growth, especially in severe oligodontia cases. The PPE device we propose assures function and esthetics in the long-term. It enhances stimulation in a passive way that leads to physiological growth of the palatal suture.

## CLINICAL SIGNIFICANCE

### Why this paper is important to the pediatric dentistry?

Pediatric dentists should be aware about the importance of maxillary transversal growth in severe oligodontia cases.

In HED cases, pediatric dentists should be capable to choose the best way in restoring the mouth that allows function, esthetics, and growth.

### What does this paper add?

Considering the importance of growth, the device we propose is a way to prevent any interference with maxilla growth in very young children.

With the use of this simple slide system, pediatric dentists can guarantee that the treatment they propose, has the best outcome in the long-term.
